# Academy of Medicine Notes

**Published:** 1893-10

**Authors:** 


### Academy of Medicine Notes.

The Council has decided to meet monthly immediately after the close of the Section on Medicine.

Drs. Arthur E. Collins and Burt G. Johnson were elected fellows at the last meeting of the Academy.

The program for the December meeting of the Academy will be arranged by the Section on Surgery.

At the next meeting of the Section on Medicine, Dr. Krauss will read a paper on What Have the Newer Therapeutical Procedures Done for Neurology. The discussion will be opened by Dr. Putnam, followed by Dr. Crego and others.

The officers of the Section on Medicine have sent to each fellow of the Academy a circular asking for original report of cases and contributions. The program committee is composed of Drs. Rochester, Frederick, Pohl, Heath, and Potter.

The library of the Academy has received a handsome donation of a full set of the proceedings of the American Association of Obstetricians and Gynecologists. Dr. W. W. Potter, the secretary of the Association since its organization, was the donor.

The next meeting of the Section on Surgery will be held Tuesday, October 3d, with the following program: Affections of the Eye, associated with and dependent upon the Scrofulous Diathesis, Dr. Alvin A. Hubbell; Discussion by Dr. Lucien Howe, Dr. H. Y. Grant, Dr. Elmer E. Starr, Dr. B. H. Grove.

